# Needs assessment for health service design for people with back pain in a hospital setting: A qualitative study

**DOI:** 10.1111/hex.13419

**Published:** 2022-02-11

**Authors:** Edward Gorgon, Katherine Maka, Andrew Kam, Gillian Nisbet, Justin Sullivan, Gerard Regan, Fereshteh Pourkazemi, Jianhua Lin, Mahmoud Mohamed, Andrew Leaver

**Affiliations:** ^1^ Sydney School of Health Sciences, Faculty of Medicine and Health The University of Sydney Sydney Australia; ^2^ Department of Physical Therapy University of the Philippines Manila Manila Philippines; ^3^ Western Sydney Local Health District New South Wales Health Sydney Australia; ^4^ Department of Rehabilitation Therapy, Shanghai Yangzhi Rehabilitation Hospital (Shanghai Sunshine Rehabilitation Center) Tongji University School of Medicine Shanghai China

**Keywords:** assessment of healthcare needs, codesign, health services research, low‐back pain, neck pain, patient‐centred care

## Abstract

**Background:**

There is a need for effective health service solutions to provide greater structure and support for implementing evidence‐based practice in back pain care. Patient involvement in developing these solutions is crucial to increase relevance, acceptability and uptake.

**Objectives:**

To determine patients' perceived needs and barriers to best‐practice back pain care, and potential solutions to better address care needs. The study is the third in a series of needs assessment studies feeding into the ‘idea generation’ for service design in a large teaching hospital in a culturally and linguistically diverse community in metropolitan Sydney, Australia.

**Design:**

We conducted a combination of focus groups and in‐depth interviews using an interpretive description approach. We used inductive thematic analysis to identify the main themes.

**Setting and Participants:**

We purposively sampled patients with diverse characteristics from the neurosurgery and physiotherapy outpatient clinics, in particular those whose primary language was English, Arabic, Persian or Mandarin. Non‐English audio recordings were translated and transcribed by bilingual researchers.

**Results:**

There were 24 participants (focus groups = 9; individual interviews = 15) when data saturation was reached. The analysis identified three key themes with several subthemes around what service designers needed to understand in helping people with back pain in this setting: (1) *This is who I am*; (2) *It's not working for me*; and (3) *What I think I need*.

**Discussion and Conclusion:**

This study highlights that perceived unmet needs of patients are underpinned by unhelpful beliefs about the causes of and solutions for back pain, misaligned care expectations, unclear expectations of the hospital role and fragmentations in the health system. To design and implement a service that can deliver better back pain care, several solutions need to be integrated around: developing new resources that challenge unhelpful beliefs and set realistic expectations; improving access to education and self‐management resources; focusing on individualized care; using a collaborative multidisciplinary approach within the hospital; and better connecting with and directing primary health care services.

**Patient or Public Contribution:**

A consumer representative of the Western Sydney Local Health District provided input during study conceptualisation and is duly recognized in the Acknowledgements section.

## 1 INTRODUCTION

Integrating best practice recommendations for back pain into routine care is challenging given most current health systems are not particularly organized to support these recommendations.^1^ Best practice guidelines typically recommend what changes are necessary, emphasizing tailoring care to patient context and preferences and prioritizing nonpharmacological management.^2^, ^3^ These changes reflect a reconceptualisation of back pain away from a traditional biomedical model, requiring a shift in perspective and behaviour from patients and clinicians. However, these recommendations have failed to resonate in clinical practice,^4^, ^5^, ^6^, ^7^ with guidelines often criticized for not being user‐friendly^8^ and for lacking applicability and implementation guidance.^9^, ^10^ Suboptimal and delayed uptake of evidence‐based practice has important ramifications, as back pain is the leading cause of disability worldwide.^6^, ^11^, ^12^ Therefore, there is a need to develop effective health system‐ or health service‐level solutions to support the translation of best practices into routine care.

One potential solution is the design of new clinical pathways for back pain.^1^, ^6^, ^7^, ^13^ Recent studies have shown the value of clinical pathways for back pain for integrating guideline recommendations, streamlining care processes across multiple disciplines and supporting shared decision‐making.^13^, ^14^ However, all except one^14^ of these alternative pathways were created for European and North American healthcare contexts. This is important because implementation outcomes vary across settings and jurisdictions, and are therefore context‐dependent.^15^, ^16^ For example, a primary care model based on prognostic stratification of back pain (STarT Back) demonstrated positive outcomes in the United Kingdom,^17^, ^18^ but did not improve healthcare utilisation and patient outcomes in the United States.^19^, ^20^, ^21^ Adaptation to the local context is therefore crucial to implementation success.

Matching healthcare solutions to context is linked to patient factors,^15^ meaning, patient needs and preferences should be considered in the design. Scoping reviews of qualitative studies have reported recurring themes of patients' perceptions of unmet needs and expectations regarding back pain care;^22^, ^23^, ^24^, ^25^ however, few studies were set in Australia, which is the local context for the present study. Most of these studies were conducted 10 or more years ago and may not reflect current needs.^22^, ^23^, ^24^ Patients' perception of unmet healthcare needs remains an important problem leading to increased use of healthcare^26^ and rising costs.^27^ Patient involvement in the development of healthcare solutions is considered crucial in promoting relevance, acceptability and uptake,^28^, ^29^, ^30^ and therefore warranted throughout the design process.

We are currently designing multidisciplinary care for back pain that is fit‐for‐context in a large teaching hospital setting to meet the needs of a diverse community in Sydney, Australia. The hospital provides high‐quality specialized healthcare for patients with back pain, including physiotherapy, neurosurgery, rheumatology, orthopaedics and chronic pain management. Further, it provides clinical education for future healthcare workers and is a research centre that promotes clinical–academic partnerships. The service design is guided by the Sax Institute translational research framework.^31^ This study is the third in a series of needs assessment studies within the framework's ‘idea generation’^31^ and marks the start of patient involvement in a codesign process.^32^ It provides a qualitative examination of current care to complement our quantitative evaluations of service delivery.

Our earlier needs assessment studies demonstrated important issues in back pain service delivery.^33^, ^34^ The majority of referrals to the neurosurgery clinic did not require surgical treatment and would be better directed to conservative management.^33^ There were signs of poor engagement by patients with conservative management, with a 39% drop‐out and noncompletion rate for physiotherapy programmes.^34^ In contrast, the small proportion of patients who required surgery were appropriately managed, in concordance with practice guidelines.^33^ Patients referred to physiotherapy received guideline‐based, active interventions (98%),^34^ in contrast to other settings and jurisdictions.^4^, ^5^ These findings further highlight the importance of contextualisation. The hospital serves a culturally and linguistically diverse (CALD) community with areas of low socioeconomic status and health literacy.^35^ Therefore, patient involvement in the service development process requires due consideration of this community diversity.

The overall aim of the study was to explore the perspectives of patients referred for clinical services for back pain in a teaching hospital setting. The specific objectives were to: (1) determine patients' perceived needs and barriers to implementing best practice care; and (2) identify potential solutions that can be applied in service design.

## 2 MATERIALS AND METHODS

### 2.1 Ethics approval

The Western Sydney Local Health District Human Research Ethics Committee (Reference Number 2019/ETH09876) approved this study.

### 2.2 Research design and approach

This qualitative study used an interpretive description approach^36^, ^37^ to understand reality through the lens of people in their lived situations.^38^ This approach was suitable for exploring phenomena from a clinical perspective as it involved: (1) rich description of both thematic patterns and individual variations that characterized phenomena; (2) critical examination of findings in light of the discipline's existing knowledge base; and (3) end‐products that could inform clinical practice. This study applied the Consolidated Criteria for Reporting Qualitative Research for comprehensiveness and transparency in research reporting.^39^

### 2.3 Study setting

The study was conducted at the neurosurgery and physiotherapy outpatient clinics of a large publicly funded teaching hospital in metropolitan Sydney, Australia. These services receive a combined total of approximately 1000 new referrals for back pain annually. The neurosurgery clinic is a funded weekly 4‐h service where three to four neurosurgeons and neurosurgery registrars review up to 30 new and follow‐up patients. The physiotherapy clinic has seven full‐time equivalent positions covered by 14 physiotherapists. Patients referred to these clinics typically are middle‐aged and older persons, have chronic back pain and are from CALD and socioeconomically diverse backgrounds,^33^, ^34^ reflecting population ageing and diversity in Greater Western Sydney.^35^

### 2.4 Participants

Adults with back pain (>18 years of age) were purposively sampled observing maximum variation^40^ to explore the range of patients' care needs and common patterns cutting across such needs. Patients from diverse backgrounds were sought, particularly those whose primary language was one of the most prevalent in the community (English, Arabic, Persian or Mandarin).^41^ Previous studies have mostly reflected the perspectives of patients from English‐speaking backgrounds.^22^, ^24^ Patients were included if they were: (1) on the clinic waitlists; (2) attending outpatient sessions at the time (i.e., physiotherapy); or (3) formally discharged or had discontinued an episode of care. All participants provided written informed consent.

Participant recruitment was conducted from September 2019 to September 2020, with a 4‐month pause (March–June 2020) due to government‐mandated activity restrictions related to the COVID‐19 pandemic. An initial sample size of 30 was estimated from similar studies. Multiple recruitment strategies were used including active recruitment by the research team (face‐to‐face and by telephone), postal and electronic mail and posting of informational flyers in clinic waiting areas.

### 2.5 Data collection

A combination of focus groups and individual interviews was used.^42^ Data were initially gathered through three face‐to‐face focus groups (*n* = 9; English = 2, Mandarin = 1). Focus groups lasted approximately 70–80 min and were held at quiet meeting rooms away from the outpatient clinics. Data were subsequently gathered through telephone interviews of 15 additional participants (English = 5, Arabic = 5, and Persian = 5) to probe in depth into the preliminary findings from the focus groups. Telephone interviews also facilitated participation whilst observing state health advisories to minimize COVID‐19 transmission. Interviews lasted 15–45 min. Participants provided demographic data (age, sex, country of birth, spinal area affected by pain, chronicity of back pain, living situation, work status and status of episode of care) through a one‐page questionnaire in their primary language. Focus groups and interviews were conducted at the participants' preferred times. There were no repeat focus groups or interviews.

Researchers (E. G., K. M., G. N., J. S., A. L.) developed semi‐structured guide questions (Supporting Information Appendix) prefaced with an explanation that responses would be specifically used to understand how to best help people with back pain and improve service delivery at the hospital. A prototype version of the questions was piloted with five volunteer patients who attended back exercise classes at the physiotherapy department but were not part of the study. Wording of some questions was updated to improve clarity and concreteness. The guide questions were used as starting points and interviewers used additional questions to follow the participants' lead. Following the three focus groups, more targeted questions were developed and incorporated into the succeeding individual interviews.

Four bilingual research team members who were not involved in the participants' clinical care nor part of the hospitals' outpatient clinics conducted the focus groups and interviews (E. G., male; J. L., male; M. M., male; F. P., female). All were healthcare professionals (E. G., J. L., F. P., physiotherapists; M. M., clinical pharmacist) and had postgraduate qualifications and research experience (E. G., J. L., M. M., masters; F. P., PhD). Participant responses were audio‐recorded and transcribed verbatim. Non‐English language audio recordings were directly translated and transcribed in English by the bilingual researchers who led the focus group or interviews (J. L., Mandarin; M. M., Arabic; F. P., Persian). Peer debriefing with another member of the research team was conducted after each focus group and individual interview to capture participants' key messages and researchers' preliminary data interpretations. Reflexivity was observed using field notes.

### 2.6 Data analysis

The researchers applied thematic analysis using an inductive iterative approach.^43^, ^44^ Four research team members (E. G., G. N., J. S., A. L.) independently reviewed the first three transcripts, which were from the focus groups. Initial codes were identified from descriptive phrases or statements (i.e., units of meaning). Individual coding by the researchers was compared, discussed and recoded over a series of meetings to create a coding framework. Individual interviews were conducted with additional probing questions included based on the analysis of the focus group data. The subsequent transcripts were analysed using the coding framework, with codes iteratively reviewed and updated. Data collection was completed when the addition of new data from the last two interviews was managed by the coding framework without requiring further modification.^45^ The first author (E. G.) led the analysis of the interview transcripts with three senior authors (G. N., J. S., A. L.) providing independent reviews and challenging the assumptions that underpinned the interpretations. Disagreements were discussed and resolved through group consensus. Codes were categorized to generate themes and subthemes, which were iteratively reviewed, defined and named. Vivid and compelling quotations supporting the themes were identified and extracted. The transcripts and analyses were not member‐checked; however, patients were sent a synthesis of the findings as part of subsequent steps to develop service design solutions. All decisions made by the researchers were documented in an audit trail. NVivo 12 software (QSR International Pty Ltd.) was used to organize qualitative data during thematic analysis, while demographic data were analysed descriptively using IBM SPSS Statistics for Windows, Version 25.0 (IBM Corp).

## 3 RESULTS

There were 24 study participants when data collection was completed. None of the participants dropped out or withdrew from the study. Table 1 summarizes the participants' characteristics. They were aged 32–81 years (mean = 53, SD = 14) and two‐thirds were female. The majority were born outside of Australia (75%) and spoke a non‐English primary language (58%). Most were retired or not employed (75%) and one‐third lived alone. Most had chronic back pain (92%) (mean, SD duration = 80, 65 months) and one‐half experienced pain in multiple spinal regions. Most (20/24, 83%) had sought care outside of the hospital system and shared insights from care received both in the hospital and community.

**Table 1:** Participant characteristics (*n* = 24)

Characteristic	*N* (%)
Age category (years)	
30–39	5 (20.8%)
40–49	6 (25%)
50–59	4 (16.7%)
60–69	6 (25%)
70 And over	3 (12.5%)
Biological sex	
Female	16 (66.7%)
Male	8 (33.3%)
Primary language spoken	
English	10 (41.7%)
Arabic	5 (20.8%)
Persian	5 (20.8%)
Mandarin	4 (16.7%)
Country of birth	
Australia	6 (25%)
China	4 (16.7%)
Iran	4 (16.7%)
India	2 (8.3%)
Lebanon	2 (8.3%)
Syria	2 (8.3%)
Afghanistan	1 (4.2%)
Egypt	1 (4.2%)
Jordan	1 (4.2%)
Philippines	1 (4.2%)
Lived with	
Spouse/partner or family	13 (54.2%)
Friends/other people	2 (8.3%)
Alone	8 (33.3%)
Engaged in paid employment	
Yes	6 (25%)
No	18 (75%)
Chronicity of back pain^a^	
Acute	1 (4.2%)
Subacute	1 (4.2%)
Chronic	21 (91.7%)
Spinal area affected	
Cervical	2 (8.3%)
Lumbar	10 (41.7%)
Combination of spinal regions	12 (50%)
Patient status at the hospital	
Waitlisted, first episode of care	7 (29.2%)
Waitlisted, previously completed episode of care	5 (20.8%)
Currently receiving care	2 (8.3%)
Discharged, completed episode of care	5 (20.8%)
Dropped out/did not complete episode of care	5 (20.8%)

^a^ Acute = less than 6 weeks; subacute = 6–12 weeks; chronic = more than 12 weeks.^46^

Three key themes were identified from the data: (1) *This is who I am*; (2) *It's not working for me*; and (3) *What I think I need*. Within each theme, subthemes were identified (Table 2).

**Table 2:** Qualitative themes and subthemes

Theme	Subtheme
1. This is who I am	My pain impacts multiple aspects of my life
	I will try anything to relieve my pain
	My back needs ‘fixing’
2. It's not working for me	My expectations are not being met
	There are barriers to meeting my needs
3. What I think I need	This is the ‘right type of treatment’ for my pain problem
	I want reassurance and long‐term solutions
	I want to be treated as an individual

### 3.1 Theme 1: This is who I am

This theme relates to the need to understand the patients' background and experience with back pain in society. Subthemes encompass the negative impacts of back pain on multiple aspects of their life, a willingness to try anything to alleviate pain, and a sense that their spine is damaged and therefore requires fixing.

Participants frequently expressed negative impacts of back pain on their quality of life, such as loss of independence, feelings of isolation and resignation.

*Well, it's not that I don't want to go [out]. It's that I know that if they're going for a walk… well then, enjoy it because I'm not going to be able to walk that hour. Unless I want to fill up on painkillers… That's a very, very frustrating part. And yes, you do become isolated because you keep saying, ‘No, no, no’,… people give up on you and stop asking*. (Participant 1)

*[I have been] In and out of hospital… I'm over it now*. (Participant 2)

Some participants alluded to wanting to try anything to relieve their back pain.

*…you're obsessed about it [back pain]. You'll pay anything and do anything you have to do to not have to live with that back pain*. (Participant 5)

Participants narrated their experiences and needs using language that suggested beliefs linked to pathology or dysfunction. Their words indicated a strong pathoanatomical or biomechanical focus with a belief that identifying and fixing structural problems in the spine were key to alleviating their pain problem.

*I have whiplash. I have a couple of discs out in the back*. (Participant 4)

*The spine is like the core skeleton of a building. Once it is impacted, you don't feel strength in any parts of your body. You feel broken. You feel you're deteriorating*. (Participant 16)

Some participants described their condition using analogies or metaphors provided or reinforced by healthcare providers.

*He [healthcare provider] said that the muscles in your back are strong. … It [sic] just pulls your spine like the cable‐stayed bridge. Your lumbar spine has collapsed, but the muscles next to them [sic] have held your back*. (Participant 7)

### 3.2 Theme 2: It's not working for me

This theme covers participants' perceptions of how they had not experienced the type of help they believed they needed over the course of their back pain condition and encounters with the health system and many healthcare professionals. Subthemes related to their feelings about their unmet expectations and difficulties encountered when accessing services.

Some participants voiced feelings of annoyance or frustration about health services encountered over the course of their condition that they perceived to be either ineffective or not aligned with their preferences, expressed by one participant as ‘*just having bad treatments along the way… I really didn't think it* [treatments received] *was what I needed. It wasn't helping me…’* (Participant 5).

Participants communicated beliefs that delayed access to services had led to missed opportunities for treatment, which in some cases had contributed to the worsening of their condition. One participant said, ‘*I strongly believe that if my case was dealt with from the beginning, I wouldn't have reached this state of pain and immobility’* (Participant 23).

Participants identified various obstacles in meeting their needs. The obstacles related to navigating the health system, being unable to afford additional services, and availability of services. Participants described long waiting times for appointments. They expressed a need for flexibility in appointment bookings and transparent processes of communication.

*But, I had to wait for half a year for this check [of my neck and upper limb],… and wait for another six months for the MRI, and then wait for another half a year after that [for my next appointment]. When I came later, they referred me to see the neurosurgeon. Again [now], I need to wait… Caused [me] some unnecessary suffering…* (Participant 9)

Participants also believed that cost of care was a barrier to receiving the right kind of help and that they might have been helped more by privately funded services, which were outside of their financial reach.

*The other thing is the cost. I could have seen a private specialist and physio, but it [sic] was way too expensive and beyond my ability to pay*. (Participant 22)

Difficulty in accessing services and facilities for health and healthcare procedures was also identified as an important barrier to meeting their needs.

*We don't have a warm water swimming pool to just go there and do exercise and walk in it. They [healthcare providers] just tell me [to] go in the water [sic] and walk. But where?* (Participant 17)

*I think at the heart of the problem is basically there aren't enough resources, doctors, physios*. (Participant 13)

### 3.3 Theme 3: What I think I need

In this theme, participants provided their perspectives of what might help them manage their back pain condition and how a hospital‐based service might assist them. The subthemes pertained to their beliefs about what constituted ‘the right type of treatment’, wanting reassurance and long‐term solutions, and wanting to be treated as an individual.

Participants appeared to generally appreciate or value the role of physical activity and exercise as a ‘right treatment’ for back pain.

*And I think as I got a stronger, I did not feel that much pain [anymore] after my exercises. And eventually, after I finished a series of physiotherapy sessions, I became so brave… And I would feel much better*. (Participant 19)

There was a general sense of wanting to avoid medications and surgery where possible, with most participants citing their lived experiences of medical side effects or the potential negative consequences of surgery.

*How is it possible that my [healthcare provider] says there is nothing that can be done, and I can only take painkillers to cope with my pain and live my life this way? Do you know how many side effects these medications have… and none of them are effective?* (Participant 16)

*Some people say, ‘That's okay, go and have surgery’. But [I think] it's risky, you may have complications afterwards*. (Participant 18)

Many participants expressed a preference for passive treatments, particularly massage. When not offered as part of conservative care, these were perceived as missing from what they expected to receive: ‘*… it was more of them consulting* [with] *me on what to do at home and what not to do to not aggravate it* [back pain]*. I guess I was looking for more of a* [hands‐on] *treatment with results’* (Participant 12).

Some participants reported using their government‐funded healthcare allocations to access massage in private clinics. One participant narrated: ‘*… only five times* [sessions] *covered by the government is not sufficient. We need at least eight to ten sessions… If I need more massage sessions, I have to pay for them’* (Participant 24).

Psychological support was identified by some participants as a fundamental component of the help they needed, alongside the physical treatments.

*I suppose probably bring in that service [psychology]. Be understanding that it [back pain] is a draining experience—do you want to talk to somebody about it? I mean, that should be front‐of‐line for all healthcare providers these days*. (Participant 3)

Participants also provided insights on how service aspects might be organized. They emphasized wanting to be seen early and given reassurance that their pain problem was not serious. Some suggested that reassurance was particularly important if they were to be placed on a long waiting list. Participants highlighted wanting advice around what they could do to help themselves while waiting for an appointment.

Participants desired consistency of care longitudinally and across healthcare providers, expressing frustration over conflicting advice and repetitious procedures, and suggesting the need for greater collaboration amongst healthcare providers.

*… [the different healthcare providers should be] talking to each other rather than it all sort of revolving around a specialist visit*. (Participant 3)

Some participants were cognisant that their condition ‘*is not going to be cured in a day, so you need to have a long‐term plan’* (Participant 7). In line with this, they felt that they did not have an adequate follow‐up. One participant opined:

*I think there is a lack of follow‐up. They don't ask how have you been doing, do you want to come back [and] have a check, or do you want to continue?* (Participant 6)

Many participants articulated their desire for support on how to self‐help correctly and safely over the long term. One participant said, ‘*If you can set the patient* [up] *to a roadmap, where you are helping them not to come back to hospital with the same problem, so that they do not have recurring back pain, I think that's really the long‐term plan’* (Participant 13). They identified a range of ways of providing support, including reassurance around how they were performing self‐help; practicable advice and credible information resources; use of online programmes or phone technology; and clinician‐supervised peer support groups. For example, one participant suggested using informational videos on ‘*What I can do to overcome the pain. The videos would have instructions and directions on what to do and what not to do, how to move properly, and how to cope with the pain’* (Participant 20).

Many participants expressed that they valued healthcare providers who treated them as an individual, acknowledged their pain experience and understood what mattered to them. They believed that they could occasionally be lost in the health system and perceived that some of the personal‐sided healthcare was sometimes not evident. Their responses revolved around being treated with respect, listened to and shown empathy; allocated sufficient time and attention to understand the pain problem; provided with appropriately paced and tailored care; and offered relevant explanations and information. Below are some examples of what they shared:

*It [being treated as a person] means that when you went there, they listened to you, they asked you, they took notice of what you're saying. They let you do it at your own pace… Whereas if you're treated as a number—Do this, do that, there you go, next one, come in!* (Participant 1)

*… I just wish I could go back there [physiotherapy clinic] again… They were lovely. They treated me like an angel and like a princess*. (Participant 2)

*Everywhere that I go outside the hospital, there is no information on me and my health background [that I have access to and can use if I need to] … I understand that this might not even be important to them, but it is very important to me*. (Participant 15)

## 4 DISCUSSION

This qualitative study explored patient perspectives of back pain care to inform service design in a large teaching hospital in a CALD community. Most of the participants had rich experiences with respect to chronic back pain and had sought healthcare in a variety of settings. Overall, they perceived that their healthcare needs related to their back pain were not being adequately met. This is consistent with previous back pain research in Australia and other parts of the world.^22^, ^23^, ^25^, ^26^ This is not surprising given the volume of research globally reporting that treatment approaches for persistent back pain are mostly unsuccessful.^6^, ^47^ Therefore, there is a need to explore new ways of organizing and delivering care for people with back pain.^6^, ^48^ The main findings of this study suggest that perceived unmet needs of patients may be linked to unhelpful beliefs about back pain, misaligned care expectations, unclear expectations of the hospital role and fragmentations in the health system.

Our participants typically described their back pain experience with reference to radiological findings, and anatomical or biomechanical constructs, with potential negative impacts on their beliefs and expectations about management. This is consistent with a traditional biomedical view of back pain^49^, ^50^ and with prevalent beliefs in the community that back pain is necessarily the result of damage or disease.^51^, ^52^ These beliefs also might explain that a large number of patients with nonspecific chronic back pain are inappropriately referred to the hospital's neurosurgical clinic. For people with nonspecific back pain where surgical solutions are limited, a pathoanatomical bias in beliefs can create unrealistic care expectations^53^ and potentially drive unhealthy coping behaviours.^51^, ^54^ This is at odds with contemporary views that persistent and disabling back pain should be managed like other chronic health conditions, with better lifestyle choices and greater patient responsibility over their own health.^53^ Until these beliefs are challenged and addressed, it would be difficult to secure patients' active engagement in evidence‐based management.

Participants described expectations of hospital‐based services that aligned poorly with best practice for back pain, as offered by the hospital clinics. For example, many participants expected massage or hydrotherapy, which practice guidelines do not recommend.^5^ In many cases, these beliefs appeared to be reinforced by participants' experiences with private sector practitioners using publicly funded chronic care packages for low‐value passive treatments. Our previous research demonstrated good guideline‐based conservative management in the hospital, but there were observed drop‐outs and discontinuations with 39% of patients.^34^ Low‐level engagement in active self‐directed treatments might be reflective of misaligned expectations. These expectations would be important to address, as these could undermine the patient–clinician therapeutic relationship and contribute to recurring perceptions of unmet needs amongst patients. Therefore, there is a need to ensure that comprehensive information and explanations are communicated to patients to better align beliefs and ensure commitment to self‐improvement and self‐management.

Many participants also expressed a thirst for information and self‐help solutions, which aligns with practice guidelines.^2^ This highlights an opportunity to reinforce exercise‐based and education‐focused interventions as high‐value self‐help solutions. With greater action expected of patients in managing their health, proportionate support mechanisms need to be implemented.^55^ Some participants voiced concerns regarding matching exercise and information to their skills and preferences, and a need for supervision and review of their programme, as similarly articulated by patients in previous research.^24^ Self‐help support for patients^55^ would be crucial for long‐term commitment and would need to be balanced against available organisational capacity. Introducing more efficient alternatives to conventional delivery would be important, for example, developing population‐based health information and self‐help programmes, and expanding existing group interventions. In designing a new service, these need to be configured to accommodate patients with a range of language and health literacy backgrounds in the target community.^35^

The findings of this study underscore the need to develop educational resources that reframe persistent back pain through a biopsychosocial lens, as well as strategies to prevent and manage secondary disability and distress, and promote effective lifetime self‐management.^53^, ^56^, ^57^ Healthcare providers have a crucial role given their strong influence on patients' beliefs^51^ and thus need to avoid inadvertently reinforcing negative and frightening pathoanatomical constructs in discussions about diagnosis and prognosis.^58^, ^59^ Patients need to understand the natural history and biopsychosocial factors of back pain, the management choices best suited to their preferences and life situations and the advantages and limitations of conservative and surgical solutions. Good first‐line care that manages patients' beliefs and expectations regarding back pain management and prepares them to engage in long‐term behaviour change would be critical to integrate into the design of a new service.

Some participants perceived being treated more as a number than as an individual person in their various encounters with healthcare, potentially undermining motivation and engagement. Participants used both positive and negative experiences of care to illustrate how they valued being treated as a person, highlighting the importance of healthcare providers' communication and interpersonal skills.^22^, ^60^, ^61^, ^62^, ^63^, ^64^ Patients who are provided validation and appropriately enabled are more likely to demonstrate autonomous motivation and positive behaviour change, potentially positively impacting health outcomes.^65^, ^66^, ^67^ These skills are key to understanding the patient's pain condition and gaining their trust and respect before healthcare providers can help them shift their beliefs and expectations and set realistic, individualized goals within a shared decision‐making process.^68^, ^69^ A large and busy hospital setting can be perceived as sterile and impersonal, despite delivering high‐quality care. There is a need to make patient‐centred care more evident and therefore recognized by patients.

Many participants perceived the health system to be fragmented, suggesting a need for improved coordination between the hospital and community, and a collaborative multidisciplinary team approach within the hospital. There were also diverse expectations about the role of the hospital in looking after back pain, with many turning to the hospital for primary healthcare services, which they are unable to afford in the community. There is clearly a role for specialized surgical and medical treatments where appropriate; however, patients who need these are the minority.^33^, ^34^ There is a need to promote greater awareness amongst primary healthcare providers on initiating timely, adequate conservative treatment and seeking pain management to assist effective coping amongst patients. The participants were reflective of the back pain cases referred to the hospital,^33^, ^34^ most of which were complex and chronic and often required long‐term solutions and multimodal management in the community.^70^ There is a need to visualize the ideal hospital service and the changes that can be implemented realistically under the existing policies and health service structure and resources. These changes need to be informed by insights from this study and accepted by patient stakeholders through a codesign process involving iterative consensus building.

## 5 CONCLUSION

This study represents the essential consumer perspective to complete the ‘idea generation’ that will feed into the design of service delivery for back pain in an Australian teaching hospital setting. Our participants were from CALD backgrounds and provided diverse experiences and perceptions across different levels of clinical care. The findings suggest the need to develop resources that challenge unhelpful beliefs and set realistic expectations, improve access to education and self‐management resources, focus on individualized care, use a collaborative multidisciplinary approach within the hospital and integrate better with primary healthcare services. The next steps require codesigning the solutions with the various stakeholders, including continual engagement with patient stakeholders and careful balancing of stakeholder needs with the organisation's goals, expectations and capacity.

## CONFLICT OF INTERESTS

The authors declare that there are no conflict of interests.

## AUTHOR CONTRIBUTIONS

Edward Gorgon, Katherine Maka, Andrew Kam, Gillian Nisbet, Justin Sullivan and Andrew Leaver conceived and designed the study. Edward Gorgon, Gerard Regan, Fereshteh Pourkazemi, Jianhua Lin and Mahmoud Mohamed carried out participant recruitment and data collection, with oversight from Katherine Maka, Andrew Kam and Andrew Leaver. Edward Gorgon, Gillian Nisbet, Justin Sullivan and Andrew Leaver provided data analysis and interpretation, with input from Katherine Maka and Andrew Kam in data interpretation. Edward Gorgon led the writing of the manuscript. All authors provided critical input in the drafting, reviewing and revising of the manuscript and approved the final version submitted for publication.

## Supporting information

Supporting information.

## Data Availability

The focus group and interview data (transcripts) that support the study conclusions are unavailable for public access because informed consent to share the complete transcripts outside of the research team was not obtained from the study participants.

## References

[1] Traeger AC , Buchbinder R , Elshaug AG , Croft PR , Maher CG . Care for low back pain: can health systems deliver? Bull World Health Organ. 2019;97:423‐433. 31210680 10.2471/BLT.18.226050 PMC6560373

[2] Lin I , Wiles L , Waller R , et al. What does best practice care for musculoskeletal pain look like? Eleven consistent recommendations from high‐quality clinical practice guidelines: systematic review. Br J Sports Med. 2020;54:79‐86. 30826805 10.1136/bjsports-2018-099878

[3] Corp N , Mansell G , Stynes S , et al. Evidence‐based treatment recommendations for neck and low back pain across Europe: a systematic review of guidelines. Eur J Pain. 2021;25:275‐295. 33064878 10.1002/ejp.1679 PMC7839780

[4] Kamper SJ , Logan G , Copsey B , et al. What is usual care for low back pain? A systematic review of health care provided to patients with low back pain in family practice and emergency departments. Pain. 2019;161:694‐702. 10.1097/j.pain.0000000000001751 31738226

[5] Zadro J , O'Keeffe M , Maher C . Do physical therapists follow evidence‐based guidelines when managing musculoskeletal conditions? Systematic review. BMJ Open. 2019;9:e032329. 10.1136/bmjopen-2019-032329 PMC6797428 31591090

[6] Foster NE , Anema JR , Cherkin D , et al. Prevention and treatment of low back pain: evidence, challenges, and promising directions. Lancet. 2018;391:2368‐2383. 29573872 10.1016/S0140-6736(18)30489-6

[7] Buchbinder R , Underwood M , Hartvigsen J , Maher C . The Lancet series call to action to reduce low value care for low back pain: an update. Pain. 2020;161:S57‐S64. 33090740 10.1097/j.pain.0000000000001869 PMC7434211

[8] Baiardini I , Braido F , Bonini M , Compalati E , Canonica GW . Why do doctors and patients not follow guidelines? Curr Opin Allergy Clin Immunol. 2009;9:228‐233. 19390434 10.1097/ACI.0b013e32832b4651

[9] Lin I , Wiles LK , Waller R , et al. Poor overall quality of clinical practice guidelines for musculoskeletal pain: a systematic review. Br J Sports Med. 2018;52:337‐343. 29175827 10.1136/bjsports-2017-098375

[10] Michaleff ZA , Zadro JR , Traeger AC , O'Keeffe M , Décary S . Overcoming overuse part 2: defining and quantifying health care overuse for musculoskeletal conditions. J Orthop Sports Phys Ther. 2020;50:588‐591. 33131396 10.2519/jospt.2020.0109

[11] Cieza A , Causey K , Kamenov K , Hanson SW , Chatterji S , Vos T . Global estimates of the need for rehabilitation based on the Global Burden of Disease Study 2019: a systematic analysis for the Global Burden of Disease Study 2019. Lancet. 2021;396:2006‐2017. 33275908 10.1016/S0140-6736(20)32340-0 PMC7811204

[12] Hartvigsen J , Hancock MJ , Kongsted A , et al. What low back pain is and why we need to pay attention. Lancet. 2018;391:2356‐2367. 29573870 10.1016/S0140-6736(18)30480-X

[13] Coeckelberghs E , Verbeke H , Desomer A , et al. International comparative study of low back pain care pathways and analysis of key interventions. Eur Spine J. 2021;30:1043‐1052. 33427958 10.1007/s00586-020-06675-2

[14] Moi JHY , Phan U , de Gruchy A , et al. Is establishing a specialist back pain assessment and management service in primary care a safe and effective model? Twelve‐month results from the Back pain Assessment Clinic (BAC) prospective cohort pilot study. BMJ Open. 2018;8:e019275. 10.1136/bmjopen-2017-019275 PMC6252686 30309987

[15] Nilsen P , Bernhardsson S . Context matters in implementation science: a scoping review of determinant frameworks that describe contextual determinants for implementation outcomes. BMC Health Serv Res. 2019;19:189. 30909897 10.1186/s12913-019-4015-3 PMC6432749

[16] Morris JH , Bernhardsson S , Bird M‐L , et al. Implementation in rehabilitation: a roadmap for practitioners and researchers. Disabil Rehabil. 2020;42:3265‐3274. 30978129 10.1080/09638288.2019.1587013

[17] Murphy SE , Blake C , Power CK , Fullen BM . Comparison of a stratified group Intervention (STarT Back) with usual group care in patients with low back pain: a nonrandomized controlled trial. Spine. 2016;41:645‐652. 26630423 10.1097/BRS.0000000000001305

[18] Karlen E , McCathie B . Implementation of a quality improvement process aimed to deliver higher‐value physical therapy for patients with low back pain: case report. Phys Ther. 2015;95:1712‐1721. 26381807 10.2522/ptj.20150035

[19] Cherkin D , Balderson B , Wellman R , et al. Effect of low back pain risk‐stratification strategy on patient outcomes and care processes: the MATCH randomized trial in primary care. J Gen Intern Med. 2018;33:1324‐1336. 29790073 10.1007/s11606-018-4468-9 PMC6082187

[20] Hsu C , Evers S , Balderson BH , et al. Adaptation and implementation of the STarT Back risk stratification strategy in a US health care organization: a process evaluation. Pain Med. 2019;20:1105‐1119. 30272177 10.1093/pm/pny170 PMC6934440

[21] Delitto A , Patterson CG , Stevans JM , et al. Stratified care to prevent chronic low back pain in high‐risk patients: the TARGET trial. A multi‐site pragmatic cluster randomized trial. EClinicalMedicine. 2021;34:100795. 33870150 10.1016/j.eclinm.2021.100795 PMC8040279

[22] Chou L , Ranger TA , Peiris W , et al. Patients' perceived needs of health care providers for low back pain management: a systematic scoping review. Spine J. 2018;18:691‐711. 29373836 10.1016/j.spinee.2018.01.006

[23] Chou L , Ranger TA , Peiris W , et al. Patients' perceived needs for medical services for non‐specific low back pain: a systematic scoping review. PLoS One. 2018;13:e0204885. 30408039 10.1371/journal.pone.0204885 PMC6224057

[24] Chou L , Ranger TA , Peiris W , et al. Patients' perceived needs for allied health, and complementary and alternative medicines for low back pain: a systematic scoping review. Health Expect. 2018;21:824‐847. 29983004 10.1111/hex.12676 PMC6186543

[25] Lim YZ , Chou L , Au RT , et al. People with low back pain want clear, consistent and personalised information on prognosis, treatment options and self‐management strategies: a systematic review. J Physiother. 2019;65:124‐135. 31227280 10.1016/j.jphys.2019.05.010

[26] Ahern M , Dean CM , Dear BF , Willcock SM , Hush JM . The experiences and needs of people seeking primary care for low‐back pain in Australia. Pain Rep. 2019;4:e756. 31579850 10.1097/PR9.0000000000000756 PMC6728007

[27] Australian Institute of Health and Welfare Impacts of chronic back problems. 2016. https://www.aihw.gov.au/getmedia/9018da61-cdf0-4e3a-bd98-2508f515290d/19839.pdf.aspx?inline=true. Accessed October 19, 2021.

[28] National Health and Medical Research Council . Statement on Consumer and Community Involvement in Health and Medical Research. 2016.

[29] Nilsen ES , Myrhaug HT , Johansen M , Oliver S , Oxman AD . Methods of consumer involvement in developing healthcare policy and research, clinical practice guidelines and patient information material. Cochrane Database Syst Rev. 2006;2006:Cd004563. 10.1002/14651858.CD004563.pub2 PMC6464810 16856050

[30] Anderst A , Conroy K , Fairbrother G , Hallam L , McPhail A , Taylor V . Engaging consumers in health research: a narrative review. Aust Health Rev. 2020;44:806‐813. 32780985 10.1071/AH19202

[31] Sax Institute Translational Research Framework: testing policy, program and service innovation. 2016. https://www.saxinstitute.org.au/wp-content/uploads/Translational-Research-Framework.pdf. Accessed October 19, 2021.

[32] Dimopoulos‐Bick TL , O'Connor C , Montgomery J , et al. “Anyone can co‐design?”: a case study synthesis of six experience‐based co‐design (EBCD) projects for healthcare systems improvement in New South Wales, Australia. Patient Exp J. 2019;6:15‐104.

[33] Hancock M , Maka K , Gorgon, E , et al. Current practice in health service delivery in a hospital neurosurgical clinic: a retrospective file review. Westmead Research Showcase. September 11, 2019. Sydney, Australia.

[34] Gorgon E , Maka K , Regan G , et al. Redesigning care for spinal pain in an Australian hospital setting: a service evaluation to identify need for change. World Physiotherapy Congress. April 9, 2021. 10.1002/msc.1695 36069172

[35] Nepean Blue Mountains, South Western Sydney, and Western Sydney Local Health Districts . Position Paper 2019: partnership for the Development of Greater Western Sydney Health, pp. 1‐22.

[36] Thorne S , Kirkham SR , MacDonald‐Emes J . Interpretive description: a noncategorical qualitative alternative for developing nursing knowledge. Res Nurs Health. 1997;20:169‐177. 9100747 10.1002/(sici)1098-240x(199704)20:2<169::aid-nur9>3.0.co;2-i

[37] Merriam SB , Grenier RS . Qualitative Research in Practice: Examples for Discussion and Analysis. 2nd ed. San Francisco, CA: Jossey‐Bass; 2019;33‐86.

[38] Weaver K , Olson JK . Understanding paradigms used for nursing research. J Adv Nurs. 2006;53:459‐469. 16448489 10.1111/j.1365-2648.2006.03740.x

[39] Tong A , Craig J , Sainsbury P . Consolidated Criteria for Reporting Qualitative Research (COREQ): a 32‐item checklist for interviews and focus groups. Int J Qual Health Care. 2007;19:349‐357. 17872937 10.1093/intqhc/mzm042

[40] Palinkas LA , Horwitz SM , Green CA , Wisdom JP , Duan N , Hoagwood K . Purposeful sampling for qualitative data collection and analysis in mixed method implementation research. Adm Policy Ment Health. 2015;42:533‐544. 24193818 10.1007/s10488-013-0528-y PMC4012002

[41] Australian Bureau of Statistics . Census summary data: cultural and language diversity fact sheet; 2016. https://www.abs.gov.au/websitedbs/d3310114.nsf/home/Census+Data+Seminars/$File/Western+Sydney+Fact+Sheet.pdf

[42] Lambert SD , Loiselle CG . Combining individual interviews and focus groups to enhance data richness. J Adv Nurs. 2008;62:228‐237. 18394035 10.1111/j.1365-2648.2007.04559.x

[43] Braun V , Clarke V . Using thematic analysis in psychology. Qual Res Psychol. 2006;3:77‐101.

[44] Braun V , Clarke V . Reflecting on reflexive thematic analysis. Qual Res Sport Exerc Health. 2019;11:589‐597.

[45] Varpio L , Ajjawi R , Monrouxe LV , O'Brien BC , Rees CE . Shedding the cobra effect: problematising thematic emergence, triangulation, saturation and member checking. Med Educ. 2017;51:40‐50. 27981658 10.1111/medu.13124

[46] Oliveira CB , Maher CG , Pinto RZ , et al. Clinical practice guidelines for the management of non‐specific low back pain in primary care: an updated overview. Eur Spine J. 2018;27:2791‐2803. 29971708 10.1007/s00586-018-5673-2

[47] Maher C , Underwood M , Buchbinder R . Non‐specific low back pain. Lancet. 2017;389:736‐747. 27745712 10.1016/S0140-6736(16)30970-9

[48] Croft P , Louw Q , Briggs AM . Transforming back pain care—why, what, and how? Pain. 2020;161:2657‐2658. 32910101 10.1097/j.pain.0000000000001990

[49] Waddell G . Preventing incapacity in people with musculoskeletal disorders. Br Med Bull. 2006;77‐78:55‐69. 10.1093/bmb/ldl008 16968689

[50] Westman A . Musculoskeletal Pain in Primary Health Care: A Biopsychosocial Perspective for Assessment and Treatment. Örebro University; 2010.

[51] Darlow B . Beliefs about back pain: the confluence of client, clinician and community. Int J Osteopath Med. 2016;20:53‐61.

[52] Hall A , Coombs D , Richmond H , et al. What do the general public believe about the causes, prognosis and best management strategies for low back pain? A cross‐sectional study. BMC Public Health. 2021;21:682. 33832463 10.1186/s12889-021-10664-5 PMC8028215

[53] Lewis J , O'Sullivan P . Is it time to reframe how we care for people with non‐traumatic musculoskeletal pain? Br J Sports Med. 2018;52:1543‐1544. 29941618 10.1136/bjsports-2018-099198

[54] Bunzli S , Smith A , Watkins R , Schütze R , O'Sullivan P . What do people who score highly on the Tampa Scale of Kinesiophobia really believe?: a mixed methods investigation in people with chronic nonspecific low back pain. Clin J Pain. 2015;31:621‐632. 25167327 10.1097/AJP.0000000000000143

[55] May CR , Eton DT , Boehmer K , et al. Rethinking the patient: using Burden of Treatment Theory to understand the changing dynamics of illness. BMC Health Serv Res. 2014;14:281. 24969758 10.1186/1472-6963-14-281 PMC4080515

[56] Lewis JS , Stokes EK , Gojanovic B , et al. Reframing how we care for people with persistent non‐traumatic musculoskeletal pain. Suggestions for the rehabilitation community. Physiotherapy. 2021;112:143‐149. 34102533 10.1016/j.physio.2021.04.002

[57] Hutting N , Johnston V , Staal JB , Heerkens YF . Promoting the use of self‐management strategies for people with persistent musculoskeletal disorders: the role of physical therapists. J Orthop Sports Phys Ther. 2019;49:212‐215. 30931733 10.2519/jospt.2019.0605

[58] Friedman DJ , Tulloh L , Khan KM . Peeling off musculoskeletal labels: sticks and stones may break my bones, but diagnostic labels can hamstring me forever. Br J Sports Med. 2021;55:1184‐1185. 33958389 10.1136/bjsports-2021-103998 PMC8551971

[59] Rajasekaran S , Dilip Chand Raja S , Pushpa BT , Ananda KB , Ajoy Prasad S , Rishi MK . The catastrophization effects of an MRI report on the patient and surgeon and the benefits of ‘clinical reporting’: results from an RCT and blinded trials. Eur Spine J. 2021;30:2069‐2081. 33748882 10.1007/s00586-021-06809-0

[60] Touloumakos AK . Expanded yet restricted: a mini review of the soft skills literature. Front Psychol. 2020;11:2207. 33013574 10.3389/fpsyg.2020.02207 PMC7500090

[61] O'Sullivan P . It's time for change with the management of non‐specific chronic low back pain. Br J Sports Med. 2012;46:224‐227. 21821612 10.1136/bjsm.2010.081638

[62] Chi‐Lun‐Chiao A , Chehata M , Broeker K , et al. Patients' perceptions with musculoskeletal disorders regarding their experience with healthcare providers and health services: an overview of reviews. Arch Physiother. 2020;10:17. 32983572 10.1186/s40945-020-00088-6 PMC7517681

[63] Laerum E , Indahl A , Skouen JS . What is “the good back‐consultation”? A combined qualitative and quantitative study of chronic low back pain patients' interaction with and perceptions of consultations with specialists. J Rehabil Med. 2006;38:255‐262. 16801209 10.1080/16501970600613461

[64] Holopainen R , Piirainen A , Heinonen A , Karppinen J , O'Sullivan P . From “non‐encounters” to autonomic agency. Conceptions of patients with low back pain about their encounters in the health care system. Musculoskeletal Care. 2018;16:269‐277. 29327405 10.1002/msc.1230

[65] Ryan RM , Deci EL . Overview of self‐determination theory: an organismic dialectical perspective. Deci EL , Ryan RM , Handbook of Self‐Determination Research. Vol 2. Rochester, NY: The University of Rochester Press; 2002:3‐33.

[66] Ng JY , Ntoumanis N , Thøgersen‐Ntoumani C , et al. Self‐determination theory applied to health contexts: a meta‐analysis. Perspect Psychol Sci. 2012;7:325‐340. 26168470 10.1177/1745691612447309

[67] Ferreira PH , Ferreira ML , Maher CG , Refshauge KM , Latimer J , Adams RD . The therapeutic alliance between clinicians and patients predicts outcome in chronic low back pain. Phys Ther. 2013;93:470‐478. 23139428 10.2522/ptj.20120137

[68] Hoffmann TC , Lewis J , Maher CG . Shared decision making should be an integral part of physiotherapy practice. Physiotherapy. 2020;107:43‐49. 32026834 10.1016/j.physio.2019.08.012

[69] Babatunde F , MacDermid J , MacIntyre N . Characteristics of therapeutic alliance in musculoskeletal physiotherapy and occupational therapy practice: a scoping review of the literature. BMC Health Serv Res. 2017;17:375. 28558746 10.1186/s12913-017-2311-3 PMC5450083

[70] The Lancet . Rethinking chronic pain. Lancet. 2021;397:2023. 34062132 10.1016/S0140-6736(21)01194-6

