# Supplementary material for: Needs assessment for health service design for people with back pain in a hospital setting: A qualitative study
Source: Health Expect. 2022 Feb 11;25(2):721–31. doi: 10.1111/hex.13419 (PMC8957737; doi:10.1111/hex.13419)
Supplement: Supplementary file 1 — Supporting information. [file HEX-25--s001.docx]

**Appendix** Guide questions for focus groups and individual interviews

- Please tell us your name and a little bit about your back and/or neck problem.
- The next few questions will be about your experiences around your back and/or neck pain:
  - What’s the most frustrating thing about back and/or neck pain?
  - How are you dealing with your back and/or neck pain?
  - If there’s something you could change about your back and/or neck pain, what would that be?
  - What if there’s no cure, what’s the next best thing?
- The next few questions will be about the types of support that people with back and/or neck pain can benefit from.
  - What’s the most helpful thing you can give to someone with back and/or neck pain?
  - That thing you wanted to change about your back and/or neck pain – what’s the best way to help?
  - What other forms of support do you think people with back and/or neck pain need (not necessarily hospital-based)?
- If you had one minute to talk to healthcare providers, what would you tell them regarding how they can best help people with back and/or neck pain?
  - If you are able to switch places with them, what would you say or do to best help patients with back and/or neck pain?
- Additional targeted questions used in the telephone interviews
  - If the participant mentions problematic aspects of care that were already identified earlier in the focus groups (for example, treated as a number and lack of access):
    - “Treated as a number” also came up several times in our discussions with other patients. What aspects made you say that/feel that way? What would help solve that?
    - Other patients have also mentioned lack of access to services. What are the ways to provide more access? Are there practical solutions you can suggest – what are those?
  - If the participant is unable to be more specific with their answers or seems to be saying the same things over and over:
    - Offer key issues already identified in the focus groups, for example, long waiting times, lack of coordination or continuity of care, cost of care, access to services, etc – Has anyone had a problem like this as well? What would help solve that?
